# Central Dental Association of Northern New Jersey

**Published:** 1887-05

**Authors:** 


### CENTRAL DENTAL ASSOCIATION OF NORTHERN NEW JERSEY.

Reported for the Independent Practitioner.

A regular meeting of the Association was held in Newark, N. J., on the evening of Dec. 20, 1886. After the transaction of the usual routine business the President, Dr. B. F. Luckey, called upon Dr. W. C. Barrett, of Buffalo, N. Y., for the paper of the evening, "The Elements of Success." (See page 235.)

#### DISCUSSION.

*President Luckey*—Gentlemen, you have heard the reading of this delightfully interesting paper. It is now open for any remarks you may have to make upon it.

*Dr. Watkins*—Mr. President, Dr. Barrett in his preliminary remarks said that he did not know whether his paper would be instructive or not, and I want to say that I think it is instructive, and that I am extremely glad that he presented this subject. I wrote a paper upon it myself at one time, which now I am ashamed to think of. It was then classed as rather elementary, and now, after hearing the paper of this evening, I am more than ever convinced that it was a school-boy production. This paper is the finest thing I have ever heard on the subject. It is nearly perfect. I move you, Mr. President, that we return a vote of thanks to Dr. Barrett for this most admirable paper.

Dr. Watkins' motion was carried.

*Dr. Pinney*—Mr. President, I do not see how there can be much discussion over this paper. It strikes me that its statements are so perfectly true and so well put that we cannot find any fault with it if we desired to. I only rise to commend it, and to say that we cannot but accept as true what he has so delightfully said. It is a pity that a greater proportion of dentists in practice do not follow the course that has been marked out in the paper, both in their manner of practice and in their connection with societies. We see many dentists' offices that are in such a positively filthy condition that it is incomprehensible how they can work themselves, or how their patients can tolerate it. If more cleanly habits were cultivated and the medicine bottles kept shut up, I think our patients would not accuse us so often of smelling like dentists.

*Dr. Dean*—Mr. President, it is quite true that there can be no discussion of this paper. Nothing can be said, because the subject is exhausted. But I would like to mention one incident in my own knowledge, that may perhaps clinch a point or two made in the paper. A dentist in my town has lost a number of patients, simply because his finger-nails were neglected. One lady told me she passed his house and saw him leaning with his hands on his front gate, and although she was intending to go to him for some work, when she saw his finger-nails she changed her mind; she never could go near him. Yet I know that man is a good operator.

*Dr. Atkinson*—One defect in the paper is that it does not sufficiently show the importance of having your intellect well stored with all that has been said, and then giving it out of your affections, not through so much of rule, but just as occasion may demand. When you say you love your neighbor, do not say, "Oh, God! how I love you!" but just go right at it. And if we love our profession, that will be the way we will do it. To do the best we can for our patients, and that is what we are required to do, demands all our understanding under the highest illumination. But do not have so much paraphernalia and so many rules and systems; just obey your interior impulses, and the law of your own sense of justice. It is not the head but the heart that makes the dentist, and that guides all the mechanism and all the ins and outs of his operations. Dr. Barrett knows what he has said there, and he must have made a good many mistakes to find it out.

*Dr. Stockton*—Mr. President, I am very glad indeed to hear this admirable paper from our excellent friend, Dr. Barrett, to-night. It is true that there are not two sides to such a question. It is also equally true that what is one man's success, or one man's ideal, is a source of failure to another. We start out in life more or less fully equipped for our work, and we succeed or we do not. The success of one man, according to his ideal, would be complete failure to another. I do not believe in any such nonsense as that the affections of your heart make you a good dentist. It cannot be done in that way. And Dr. Barrett left out of his list of requirements *the* element of success. I do not care if a man has the affections of the world in his heart, I do not care if he is the most polite man that ever lived and wrote a book on politeness, he will not succeed as a dentist unless he knows how to do good dentistry.

*Dr. Osmun*—Mr. President, it seems to me that the gist of this paper is simply consecration. The act of consecration is but a moment; the fulfillment of the consecration is a life's work. I believe also in natural ability. We have natural musicians, natural artists, natural mechanics, and natural this, that, and the other. I do not believe there was ever a man who did not have a natural talent for art who became an artist. He may learn to mix colors and apply them by given rules, but that does not make him an artist. Neither do I believe that any man who has not music in his soul will ever make a musician, in any proper sense of the word, no matter how excellent a teacher he may have or how long he may study and practice. And so I believe that some natural ability is necessary to make a dentist. I have, in my little experience with different students in my office, observed that the one who has natural ability acquires things rapidly and accurately, and does not have to be told again and again to do the same thing; and this is because he has a natural mechanical turn of mind. Another student never learns anything easily, but must be shown over and over again, and every case he makes precisely like the one preceding. Such an one will never be successful. He may be honest, may have the love of humanity in his heart, may be kind and sympathetic, may have the politeness of a Chesterfield, and yet be a failure as a dentist. Possibly he might make a fine orator, a good physician, an excellent carpenter, a successful farmer, or fruit raiser, but he cannot be a dentist. It requires a certain combination of natural talents which can never be obtained; they are given to man by his Creator, and he must develop them by study and practice, and by actual every-day work. Then when he has gone, there will be a good man gone, and a good dentist gone.

*Dr. Kingsley*—I listened, Mr. President, with a great deal of interest to the paper that has been read and the remarks that have been made upon it; and though some of the speakers have said that the essayist had exhausted the subject, and there was little or nothing to be said, no criticism to be made of the paper, but only commendation to be offered, yet while it was going on I was revolving in my mind one sentence that was used, and that was referred to indirectly by our friend Osmun. The statement in substance was that there was no such thing as genius; that what passed for that was simply industry. Gentlemen, that statement of Dr. Barrett is simply bosh. He doesn't mean exactly that either.

*Dr. Barrett*—Yes he does mean it.

*Dr. Kingsley*—Then I am sorry for him, because there is such a thing as genius quite irrespective of industry. There is a patient industry of the most untiring kind that never accomplishes the object in view at all. You must have natural faculties, or predisposition, or peculiar temperament, or what is called genius. Take two persons, one having simply patient industry, and the other possessed of this genius, and I leave it to any of you to choose which of the two you would prefer to make a dentist out of. It is not necessary for me to decide that question.

*Dr. Barrett*—Will you define genius?

*Dr. Kingsley*—I have already defined it as consisting of natural talent or faculties, or predisposition to the accomplishing of certain objects. I do not think it is necessary to go into the definitions of the books; I think you understand perfectly what I mean. It is said, I think with a great deal of truth, that no great novelist or author who has excelled in portraying human character has ever been able to make a hero or heroine greater than himself or herself; that their hero is always a picture of self—the ideal character that they present is their own character.

*Dr. Barrett*—Mr. President, if there be any bosh in the remarks I have made concerning genius, the acme of bosh would seem to be Dr. Kingsley's definition of the term. I restricted the meaning by the word "intuitive," and in this sense genius is supposed to be that innate knowledge which does not come from study, or from industry, or from perseverance; it is understood to be the natural qualification, the inborn skill that is inherent in the man, and he who is endowed with genius is supposed, in the common acceptation of the term, to be one who is beyond the necessity for the study which is indispensable to most men. I do not believe such a man exists. I do not believe men are endowed with the instinct of the birds of the air, that are capable of building their first nest as perfectly as those which they build afterwards. We know very well that the men who have made the greatest success as artists, lawyers, orators, sculptors, musicians, have been men of untiring industry. If Dr. Kingsley had pursued the calling of a sculptor, he himself might have attained no inconsiderable fame in the world of art. He has shown some talent in that field already, but I do not think he will claim to be endowed with such genius as would enable him, the first time he attempts it, to present the acme of all the conceptions and ideas which were in his mind. I think he will say that if he were to set himself at work and study for some years in that direction with his usual industry, he himself could improve upon himself. Genius, as I understand it, is that which places a man beyond the necessity of study. I do not think such a man exists upon the face of the globe.

I certainly hold that young men are not all endowed with equal ability in any particular line. Some are naturally more quick of perception than others. There is such a thing as talent in, and adaptability for, any special work. But talent, ability, capacity and aptitude are widely different from the intuitive genius to which reference is made in the paper. I do not believe that any man is endowed with a special intuition that will take the place of faithful study and hard work.

*Dr. Atkinson*—We are dealing with principles. What are they? To define a principle puts the best definer to his trumps. What is genius? It is the inherited possibilities and molecular experience of ancestral endowment, or else civilization does not amount to anything. There is registered in the organism of individuals a tendency to see clearly and know; they are endowed, not with knowledge, not with all the paraphernalia of knowledge, but with educational faculties that make them seers. I was brought up in the church, believing that the clergy really had what they pretended to have, the divine light of wisdom ; and when I found they had been feeding me taffy, I was all broken up. I had put my trust in that which was not to be trusted. Where then shall we put our trust? We must learn to take things as we go. That is what ails Dr. Kingsley and myself; we live in the present, and we feel as young as if we were boys. We are boys in our affections and purposes. As was wisely said in the paper, read all the journals. It may be that you slip some grain through the meshes of the sieve, but it will not all escape, and you will gain some advantage.

*President Luckey*—I would like to ask Dr. Barrett what he would call Blind Tom, with his peculiar musical talent; is that genius ?

*Dr. Barrett.*—I believe, Mr. President, that there is no law which is not proved by its exceptions. It has been so stated more than once. I might retort upon the President, if I thought he asked the question seriously, by asking him if music, which has been called divine, should not be esteemed the lowest and most debased of all the callings to which man's attention may have been directed because an idiot may excel in it. Blind Tom was possessed of musical aptitude, but he was forced to learn his instrument as well as another. That he learned with greater facility was largely because his whole soul and being was devoted to and absorbed by it, through the love which he had for it. If another was as much enamored of dentistry he could not avoid excelling in it.

After the close of the discussion the essayist exhibited some specimens of prehistoric dentistry and other objects of interest to professional eyes. He said: Those who have read the INDEPENDENT

Practitioner are aware that Dr. Van Marter, of Rome, Italy, has made an exhaustive study of ancient dentistry, a matter of interest to every dentist who has the welfare of his profession at heart. It has been supposed that dentistry was a modern art or science. It is not at all modern, so far as the mechanical portion of it goes. The ancient Etrurians, who have given their name to Etruscan gold, are represented to us as being a very wonderful people. All of our records of them are obtained from their graves, and are necessarily few because of their custom of cremating their dead. But persons of distinction were sometimes buried in elaborate tombs. Some of these graves have been recently discovered, and Dr. Van Marter was enabled to secure the contents of one. It was the custom of that people to bury with their dead the treasures dearest to them, and those things that in life were held most valuable, as many of the barbarous nations of the earth have sacrificed that which was most prized by those who had passed away. In this way they put into the graves of their dead the precious records of the arts in their time, and happily for us the opening of these graves has revealed to us the exact condition of the mechanic arts of that day. We have not been able to decipher their language, specimens of which are found; but I am going to show you some specimens of their dentistry. I hold in my hand two pieces of dental art that are two thousand five hundred years old, that date back to about the time of the supposed founding of Rome, seven hundred years before the Christian era, and which are among the earliest relics discovered. Here are three lower teeth, a bicuspid, a cuspid, and a central incisor, which are held together by a band of pure gold, not soldered, but beaten out of a solid piece of gold, the bands enclosing these three teeth all being connected by a bar of the same metal. The whole piece seems to have been made for the purpose of holding in position one of these teeth that had been implanted, or loosened by disease. There is also an upper incisor with an artificial crown. Here are also some decayed teeth from the same grave, that will show you that caries was as rife two thousand five hundred years ago as it is to-day. There are also some gold spiral ornaments for the hair, of exquisite workmanship. These teeth are excessively frail, and probably would fall in pieces if they were dropped upon the floor, but I am going to do what I have never done before in a society, pass them around for your examination. Gentlemen, you are looking upon the very earliest records of dentistry of which the world has any positive knowledge, and it is to the liberality of a New York dentist, who paid the purchase money, that you are indebted for this privilege.

